# Supplementary material for: Association between oral microbiome and seven types of cancers in East Asian population: a two-sample Mendelian randomization analysis
Source: Front Mol Biosci. 2023 Nov 21;10:1327893. doi: 10.3389/fmolb.2023.1327893 (PMC10702768; doi:10.3389/fmolb.2023.1327893)
Supplement: Supplementary file 1 [file DataSheet1.ZIP › Supplementary Materials/MR plots of saliva/saliva═╝/Breast cancer/pheno.3203_to_breast cancer_forest.pdf]

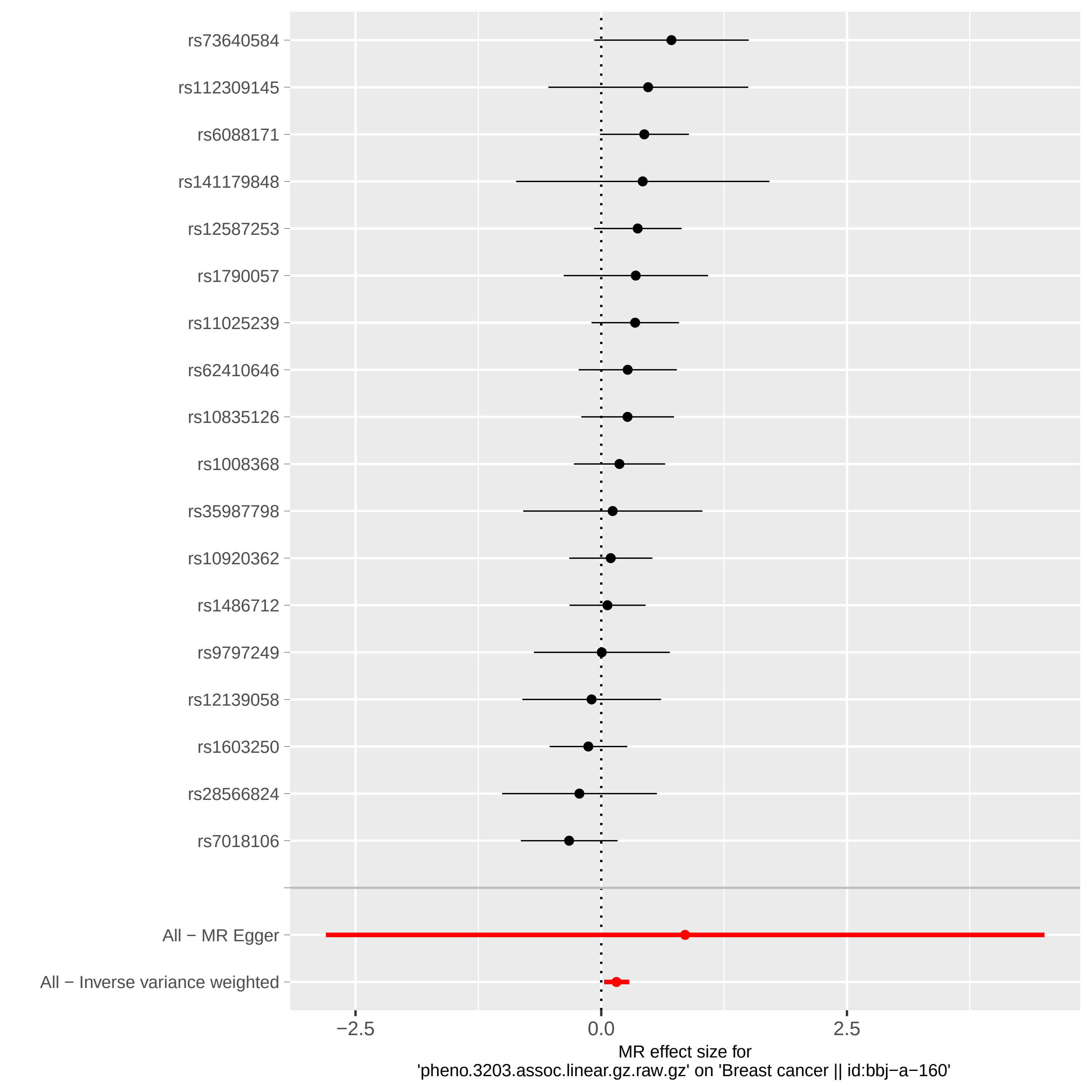
rs73640584
rs112309145
rs6088171
rs141179848
rs12587253
rs1790057
rs11025239
rs62410646
rs10835126
rs1008368
rs35987798
rs10920362
rs1486712
rs9797249
rs12139058
rs1603250
rs28566824
rs7018106
All − MR Egger
All − Inverse variance weighted
−2.5
0.0
2.5
MR effect size for
'pheno.3203.assoc.linear.gz.raw.gz' on 'Breast cancer || id:bbj−a−160'
